# Enabling multiscale modeling in systems medicine

**DOI:** 10.1186/gm538

**Published:** 2014-03-21

**Authors:** Olaf Wolkenhauer, Charles Auffray, Olivier Brass, Jean Clairambault, Andreas Deutsch, Dirk Drasdo, Francesco Gervasio, Luigi Preziosi, Philip Maini, Anna Marciniak-Czochra, Christina Kossow, Lars Kuepfer, Katja Rateitschak, Ignacio Ramis-Conde, Benjamin Ribba, Andreas Schuppert, Rod Smallwood, Georgios Stamatakos, Felix Winter, Helen Byrne

**Affiliations:** 1 Department of Systems Biology & Bioinformatics, University of Rostock, 18051 Rostock, Germany; 2 Stellenbosch Institute for Advanced Study, Wallenberg Research Centre at Stellenbosch University, 7600 Stellenbosch, South Africa; 3 European Institute for Systems Biology & Medicine, CNRS Institute of Biological Sciences, Claude Bernard University, Université de Lyon, 69100 Villeurbanne, France; 4 Sanofi Pasteur, 69280 Marcy l’Etoile, France; 5 INRIA Paris-Rocquencourt, Laboratoire Jacques-Louis Lions and Institut Universitaire du Cancer, UPMC, 75005 Paris, France; 6 INRIA Paris - Rocquencourt, Domaine de Voluceau-Rocquencourt, 78153 Le Chesnay, France; 7 UPMC University of Paris 06, CNRS UMR 7598, Laboratoire Jacques-Louis Lions, 75005 Paris, France; 8 Centre for Information Services & High Performance Computing, Technical University Dresden, 01062 Dresden, Germany; 9 Interdisciplinary Centre for Bioinformatics (IZBI), University of Leipzig, 04109 Leipzig, Germany; 10 Department of Chemistry, University College London, London WC1H 0AJ, UK; 11 Department of Mathematical Sciences, Politecnico di Torino, 10129 Torino, Italy; 12 Wolfson Centre for Mathematical Biology, University of Oxford, Oxford OX2 6GGUK, UK; 13 Institute of Applied Mathematics, Interdisciplinary Centre for Scientific Computing and BIOQUANT, University of Heidelberg, 69120 Heidelberg, Germany; 14 Computational Systems Biology, Bayer Technology Services GmbH, 51368 Leverkusen, Germany; 15 Institute of Applied Microbiology, RWTH Aachen, 52056 Aachen, Germany; 16 IMACI, Instituto de Matemática Aplicada a la Ciencia y la Ingeniería, Universidad de Castilla la Mancha, 13071 Ciudad Real, Spain; 17 NuMed, Ecole Normale Supérieure de Lyon, 69342 Lyon, France; 18 Technology Development, Bayer Technology Services GmbH, 51368 Leverkusen, Germany; 19 Joint Research Centre for Computational Biomedicine, RWTH Aachen University, 52056 Aachen, Germany; 20 Kroto Research Institute, University of Sheffield, Sheffield S10 2TN, UK; 21 In Silico Oncology Group, Institute of Communication and Computer Systems, National Technical University of Athens, 15773 Zografou, Greece; 22 ASD GmbH Rostock, 18059 Rostock, Germany; 23 School of Mathematical Sciences and Department of Computer Science, University of Oxford, Oxford OX1 3QD, UK

## From reactions in cells to organ physiology

Systems medicine is an interdisciplinary approach that integrates data from basic research and clinical practice to improve our understanding and treatment of diseases. Systems medicine can be seen as a further development of systems biology and bioinformatics towards applications of clinical relevance. The term 'systems' refers to systems approaches, emphasizing a close integration of data generation with mathematical modeling [1-3]. The (mal)functioning of the human body is a complex process, characterized by multiple interactions between systems that act across multiple levels of structural and functional organization - from molecular reactions to cell-cell interactions in tissues to the physiology of organs and organ systems. Over the past decade, we have gained detailed insights into the structure and function of molecular, cellular and organ-level systems, with technologies playing an important role in the generation of data at these different scales.

Under the umbrella of systems biology, workflows of data-driven modeling and model-driven experimentation have led to the development of computational models that describe processes at all levels, including gene regulatory networks, signal transduction pathways and metabolic networks, cell populations, structured tissues, pharmacokinetic and pharmacodynamic models that analyze drug or vaccine action at the whole organism level, and pharmacogenomic models of disease risk and drug and vaccine exposure at the patient-population level.

Major challenges exist for the development of quantitative and predictive models that span the gap between cell-level biochemical models and organism-level pharmacokinetic and pharmacodynamic models. One hurdle is the technical difficulty associated with generating sufficiently comprehensive quantitative datasets for large numbers of system variables, across different levels of organization. Another major challenge is the development of efficient tools that can handle the wide variety of available data and identify relevant data subsets from existing repositories. Overcoming these hurdles will lead to the development of models that can be used in clinical practice while accounting for the uncertainty in the data.

Another important challenge for systems medicine is the integration of this knowledge across the relevant levels of organization. Large, long-term research initiatives, like the Virtual Physiological Human, the Virtual Liver or the Human Brain Project, are aiming to develop comprehensive, computational representations of organs and organ systems. Here, we focus on opportunities for comparatively small interdisciplinary collaborations between clinicians and modelers who are targeting specific questions of clinical relevance. In the projects that we envisage, modeling is not the final goal; rather, it is a tool that can be used to advance understanding of the system, to develop more directed experiments in the laboratory and, ultimately, to generate testable predictions to enable improved therapies and prophylaxes.

## Multiscale modeling

The fields of theoretical and mathematical biology have pioneered the development of mathematical and computational models of biological systems. Systems biology has contributed workflows for data-driven modeling and model-driven experimentation to the life sciences. Taken together, these provide a considerable body of experience for modeling at different levels (Box 1).

This list is necessarily incomplete and serves here to indicate the progression of modeling efforts, with an increasing diversity of approaches available, a tendency to combine approaches in modeling workflows, and clear indications of an increasing number of successful case studies.

Multiscale modeling serves as a ‘macroscope’ that integrates the evidence available at different levels of a particular system’s structural and functional organization while enabling us to zoom in on the details where necessary. For experimental models, it is almost certain that not all parameters influencing a disease will be known and, often, known parameters cannot be influenced and/or measured. This makes it difficult to unambiguously identify the mechanisms at work in diseases. Modeling therefore makes an epistemic contribution by allowing *in silico* (simulation) experiments under defined conditions, aiding the exploration of hypotheses by explicitly accounting for the uncertainty that arises from biological complexity.

Central to these efforts are data of high quality that are quantitative, derived from reproducible studies and that cover multiple levels of functional and structural organization, generated from a range of experimental systems. A particularly important challenge is to enable clinical validation of models and simulations.

## Actions required for multiscale modeling

Our discussions have identified a number of recommendations.

### Short-term recommended actions

With respect to data collection and modeling, in the short-term (that is, the next one to two years), the following actions should be given priority:

1. Exploitation of existing data as a starting point for multiscale modeling. This will lead to the identification of gaps in data and, therefore, in understanding of underlying mechanisms, and will improve the targeted generation of new data that can be exploited for quantitative models.

2. Development of standard operating procedures and quality standards for the systematic collection of quantitative data and information. This will enable models to be built and validated for specific applications.

3. Definition of test scenarios and proof-of-concept studies.

4. Identification of required standards and ontologies (for example, a markup language and ontology for individual-based models) for models and data repositories in systems medicine.

5. Development of concepts for dedicated modeling workflows for the integration of data and models.

These research actions would benefit from initiatives that coordinate training and encourage collaborations between clinicians and multiscale modelers; support coordinated clinical projects to bring clinicians, modelers and biologists together; and run sandpits for modelers and clinicians to develop proposals to address clinical questions, to improve mutual understanding and provide realistic expectations for such collaborations.

### Medium-term recommended actions

In the medium-term of two to three years, attention should focus on the provision of suitable information technology infrastructure and the development of standards, such as:

1. The development of computational tools and algorithms for efficient multiscale simulations.

2. The development of mathematical formalism to analyze and compare multiscale models such as parameter estimation, sensitivity analysis, identifiability analysis and image analysis.

3. Support for the development of workflows for modeling, including computational tools that support data management, model construction and analysis.

4. Methods to integrate different physical phenomena, including those of mechanotransduction, electrical, mechanical and chemical origin.

5. Methods to investigate the interplay between the environment, cell behavior and cell fate.

In the medium term, the goal is to develop multiscale models of normal physiology and disease. The development of these models should be driven by clinical questions to provide the basis for clinical validation in the longer term. This will require technologies that promote and facilitate the collaboration of multidisciplinary teams.

### Longer-term recommended actions

On a timescale beyond four years, the focus should be on the application and validation of multiscale models in the clinic. To this end, it is desirable to:

1. Enhance the formation of small-scale networks focused on specific clinical needs, possibly clustered in a larger integrated project (longer timescale).

2. Have a funding model in place for small groups of two to three partners (one to two modeling postdocs, one to two experimental postdocs plus consumables and travel between laboratories). Such a funding model has been applied in systems biology to good effect, with smaller groups being integrated through networks.

The main priority for systems medicine is the development of computational models that integrate data and knowledge from the clinics and basic science (*in vitro* and animal model experiments) and are applicable to individual patients. The aim is to derive a mechanistic understanding of pathologies, prophylaxy and support of therapy optimization. This requires the development of concepts, methods and tools that support the integration of organizational levels to develop interfaces between the computational and mathematical frameworks used in systems medicine.

## Box 1. Different levels at which modeling of biological systems has been carried out

***Molecular modeling,*** for example

•Drug/vaccine target interactions

•Prediction of pathogenic mutations

•Protein-protein interactions

***Modeling of subcellular processes,*** for example:

•Signaling pathways, gene regulatory networks, metabolic networks

•Drug target prediction by sensitivity analysis

•Linking signaling pathways to phenotypic changes (for example, regression models)

***Individual-cell or cell-based models,*** for example:

•Behavior of individual cells in their microenvironment

•Cell-cell interactions

•Control of cell material properties and shape by molecular mechanisms, and the effect of physical properties of cells and their environment on cell decisions

***Tissue/organ level models,*** for example:

•Population dynamics, the formation and maintenance of tissue architecture

•Cell-stroma interactions, for example epithelial-mesenchymal transition

•Mechanobiology/biomechanical models

***Body systems level models,*** for example:

•Pharmacokinetic/pharmacodynamic modeling

•Physiologically based pharmacokinetic modeling for applications in systems pharmacology

•Modeling environmental influences and long-term clinical events (for example, overall survival).

## Competing interests

The authors declare that they have no competing interests.
